# Acoustic adaptation to city noise through vocal learning by a songbird

**DOI:** 10.1098/rspb.2018.1356

**Published:** 2018-10-10

**Authors:** Dana Lynn Moseley, Graham Earnest Derryberry, Jennifer Nicole Phillips, Julie Elizabeth Danner, Raymond Michael Danner, David Andrew Luther, Elizabeth Perrault Derryberry

**Affiliations:** 1 Department of Biology, George Mason University, Fairfax, VA 22030, USA; 2 Department of Biology, James Madison University, Harrisonburg, VA 22807, USA; 3 Smithsonian Conservation Biology Institute, Migratory Bird Center, Smithsonian Institution, National Zoological Park, PO Box 37012-MRC 5503, Washington, DC 20013-7012, USA; 4 Smithsonian Mason School of Conservation, 1500 Remount Road, Front Royal, VA 22630, USA; 5 Museum of Natural Science, Louisiana State University, Baton Rouge, LA 70802, USA; 6 Department of Ecology and Evolutionary Biology, Tulane University, New Orleans, LA 70118, USA; 7 Department of Biological Sciences, California Polytechnic State University, 1 Grand Avenue, San Luis Obispo, CA 93407, USA; 8 Department of Biology and Marine Biology, University of North Carolina Wilmington, Wilmington, NC 28403, USA; 9 Department of Ecology and Evolutionary Biology, University of Tennessee, Knoxville, TN 37996-1610, USA

**Keywords:** cultural selection, song learning, vocal performance, anthropogenic noise, birdsong, urbanization

## Abstract

Anthropogenic noise imposes novel selection pressures, especially on species that communicate acoustically. Many animals—including insects, frogs, whales and birds—produce sounds at higher frequencies in areas with low-frequency noise pollution. Although there is support for animals changing their vocalizations in real time in response to noise (i.e. immediate flexibility), other evolutionary mechanisms for animals that learn their vocalizations remain largely unexplored. We hypothesize that cultural selection for signal structures less masked by noise is a mechanism of acoustic adaptation to anthropogenic noise. We test this hypothesis by presenting nestling white-crowned sparrows (*Zonotrichia leucophyrs*) with less-masked (higher-frequency) and more-masked (lower-frequency) tutor songs either during playback of anthropogenic noise (noise-tutored treatment) or at a different time from noise playback (control treatment). As predicted, we find that noise-tutored males learn less-masked songs significantly more often, whereas control males show no copying preference, providing strong experimental support for cultural selection in response to anthropogenic noise. Further, noise-tutored males reproduce songs at higher frequencies than their tutor, indicating a distinct mechanism to increase signal transmission in a noisy environment. Notably, noise-tutored males achieve lower performance songs than their tutors, suggesting potential costs in a sexual selection framework.

## 1. Introduction

Noise from human activities is a global environmental challenge [1]. Acoustic communication is especially impacted as anthropogenic noise is often high energy at low frequencies, which can affect the perception of auditory signals by masking lower frequencies [2]. Noise-impeded communication has significant consequences for mate choice, resource defense, parental care and predator avoidance [3]. Studies in areas of relatively high anthropogenic noise report upward shifts in frequency components of vocalizations in half of the 60+ species of songbirds tested on five continents [4], as well as in frogs [5], some whales [6] and insects [7], although some of these studies may be flawed because of how frequency was measured [8]. Nevertheless, frequency shifts are documented in diverse taxa, and a key question is how these changes occur.

A recent focus of attention is on the mechanisms that enable changes in auditory signals both within an individual's lifetime and across generations [9]. One mechanism is immediate flexibility, in which adults alter their vocalizations in real time in response to noise pollution. For example, male house finches (*Carpodacus mexicanus*) shift the minimum frequency of their songs in response to increasing levels of low-frequency noise [10]. A second mechanism is genetic selection (either natural or sexual) favouring individuals that produce signal structures less masked by noise (e.g. sensory drive [11]). For example, insect noise may set the upper limit of song frequencies in Amazonian birds [12]. A third mechanism has been proposed for animals that learn their vocalizations—individuals preferentially learn signals that transmit most effectively in a given environment [13]. Applying this hypothesis to an environment with low-frequency anthropogenic noise, the prediction is individuals will preferentially learn vocalizations with higher frequencies [14]. The selective process occurs when individuals choose which vocal models to learn, and so it is the individual that acts as the selective agent, with the selective value referring to the survival and spread of the vocal model, not the Darwinian fitness of the individual [13]. Because this process is a cultural analogue of natural selection, it has been referred to as ‘psychological selection’ [15] or ‘cultural selection’ [16]; here, we use the latter term. Cultural selection occurs via multiple modes of transmission, including vertical (parent to offspring), oblique (across generations but not direct descendants) or horizontal (between members of the same generation) [16]; the latter increases the potential rate of evolution [17].

There has been extensive observational and experimental research on immediate flexibility (reviewed in [4]) and sensory drive (reviewed in [18]). In comparison, there have been few tests of the cultural selection hypothesis [9]. In one such test, juvenile male swamp sparrows (*Melospiza georgiana*) exclusively learned non-degraded songs over environmentally degraded songs [19]. This study provided good evidence for the hypothesis of cultural selection but left open the question of how anthropogenic noise impacts vocal learning. Indeed, multiple mechanisms could occur during vocal learning, such as frequency adjustments during ontogeny, which would lead to songs better suited for urban environments. A recent study on great tits (*Parus major*) suggests that exposure to city-like noise does not result in juveniles reproducing adult tutor songs at higher frequencies through ontogenetic adjustments, but this study was not designed to test cultural selection for less-masked songs [20]. Therefore, there is a limited empirical insight into one of the key mechanisms that may explain *whether* and *how* learned vocalizations change over cultural generations in response to anthropogenic noise. Addressing this gap in knowledge is important given animals that learn their vocalizations may be the most able to respond to anthropogenic noise pollution [21].

Here, we test the hypothesis that cultural selection is a mechanism of adapting to city noise for white-crowned sparrows (*Zonotrichia leucophrys nuttalli*; WCSP) collected in the field from nests in the city of San Francisco, CA, but reared in the laboratory. We assigned nestling males to two groups—experimental and control—and tutored them individually with eight different sets of stimuli. All males were also exposed to low-frequency, city-like noise—experimental males heard noise during song tutoring, which masked their tutor songs (hereafter, noise-tutored males), whereas control males heard the same duration of noise at a different time of day to control for the physiological and cognitive impact of noise exposure [22]. Both groups were tutored with the same two categories of songs: (i) lower-frequency songs, which were more masked by noise; and (ii) higher-frequency songs, which were less masked by noise. We predicted that noise-tutored males would learn the higher frequency, less-masked songs, whereas control males presented with the same songs in the absence of masking noise would learn from either category. Once males crystallized an adult song, we determined the category of tutor song copied (more or less masked) and compared tutor choices between the experimental and control groups. We then examined how males reproduced their tutors' songs, specifically testing if noise-tutored males reproduced copies at higher frequencies and at lower performance than their tutors.

## 2. Material and methods

### (a) Species

**Figure 1.**
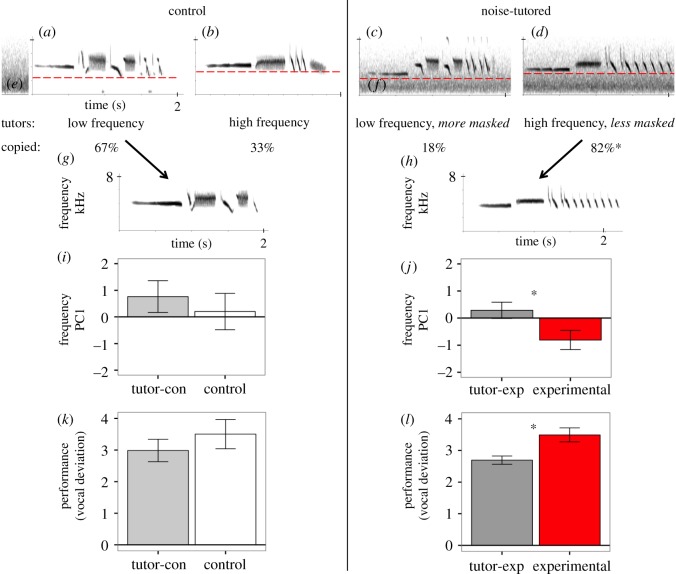
Males exposed to masking noise learn less-masked songs and sing them at higher frequencies. (*a*–*h*) Examples of white-crowned sparrow songs are depicted in spectrograms graphing frequency (0–8 kHz) against time (0–2 s). Each bird in both control (left, *n* = 6) and noise-tutored (right, *n* = 11) groups were tutored individually with three renditions of lower-frequency song types (e.g. (*a,c*)) and three renditions of higher-frequency song types (e.g. (*b,d*); see electronic supplementary material, figure S1). Birds in the control group heard city-like noise at a time separate from song tutoring ((*e*) city-like noise played separately), whereas birds in the noise-tutored group heard city-like noise that overlapped with, and thus masked, their tutor songs ((*f*) masking noise; see electronic supplementary material, figure S1 for details on masking noise). High- and low-frequency tutor songs differed by 319 Hz average minimum frequency and 24.3% average duration masked by noise measured at 10 m. The dashed line indicates the minimum frequency of each example tutor song, as measured from the amplitude spectrum at −36 dB. Control birds showed no significant difference between tutor sets for the songs they chose to copy, and four out of six (67%) copied the *lower*-frequency tutor songs (e.g. (*g*)). By contrast, 9 out of 11 (82%) noise-tutored birds copied the *higher*-frequency, less-masked tutor songs (e.g. (*h*)) significantly more often. (*i*–*l*) Control males' songs did not differ significantly from the exact tutor songs they chose to copy in frequency (PC1, (*i*)) or vocal deviation (*k*). Noise-tutored birds sang copied songs at significantly higher frequencies than the tutor songs they copied (PC1: minimum and peak frequencies load negatively) (*j*), and achieved lower performance (larger vocal deviation (*l*)). Bar plots depict mean ± s.e.m. for each respective trait, asterisks (*) here indicate significance at alpha 0.05 (see Results section and electronic supplementary material, S1). Light grey bars (tutor-con) indicate the tutor song models identified as best matched to control males’ copies, and dark grey bars (tutor-exp) indicate the tutor songs identified as best matched to noise-tutored males' copies. White bars indicate control males’ learned songs and red bars indicate noise-tutored (experimental group) males' learned songs. See electronic supplementary material, tables S2–S5 for results of statistical analyses.

The Nuttall's subspecies of white-crowned sparrows produces songs that consist of a series of notes, organized into syllables and phrases, which typically begin with a whistle and end with serially repeated syllables or notes (figure 1*a–d*). Song development follows the typical progression from a sensory phase to a sensorimotor phase, including subsong and plastic song, with a final phase of the crystallized adult song [23]. In laboratory-based song development studies, subsong begins at approximately 36 days old, plastic song at 265 days old and the median age of song crystallization is 323 days [24], at which point adults crystallize a single song type [23]. All song material learned is acquired between the ages of 20–130 days (median = 52 days) in this subspecies [24].

### (b) Subjects

Our subjects were 17 males collected in the wild as 3–9 days post-hatch nestlings from 12 nests at locations within the Golden Gate National Recreation Area (GGNRA) in San Francisco, California, USA between 13 and 24 April 2015. GGNRA is an urban park with multiple sources of anthropogenic noise including heavy traffic from the Golden Gate Bridge, construction and other human activity [25–27]. We determined the birds’ sex using DNA following standard methods [28]. Males were assigned to two groups with males collected from the same nest (5 sibling pairs) separated into different groups: 11 experimental (hereafter, noise-tutored) males and 6 control males. Males were individually housed in sound attenuation chambers (Industrial Acoustics Model Mac-1) such that they could not hear each other or the tutoring regime of other males. All birds were run through the experiment concurrently. We followed standard methods to house and hand-rear these birds [29] (see electronic supplementary material, S1).

### (c) Song recordings

To obtain tutor songs, we recorded singing territorial males in the field in five California locations (Bonny Doon, Sonoma, San Francisco, Manchester and Sans Simeon). Each location produced acoustically distinct songs. All songs were recorded using a Marantz PMD 661 digital recorder, Sennheiser ME62 omnidirectional microphone and Saul Mineroff SME–1000 parabola. We also recorded hand-reared males in the laboratory. During the subsong period, males were recorded at least twice monthly. Once plastic song began, males were recorded weekly until songs crystallized. Laboratory recordings were made using Shure SM57 unidirectional microphones, a Saffire 40 soundboard and Sound Analysis Pro [30]. We recorded all songs with a sampling rate of 44.1 kHz and stored recordings as uncompressed .wav files. We then resampled songs at 25 kHz for all subsequent sound analysis in SIGNAL v. 5 [31].

### (d) City-like noise

We measured background noise levels on 16 WCSP territories in GGNRA following methods developed in [32], see electronic supplementary material, S1 for details. We then averaged these 16 noise spectra and generated noise in Reaper v. 4.76 [33] to mimic this noise spectrum by applying an FFT filter to white noise, which decreased the spectral energy by 6 dB per octave up to 2.5 kHz and 9 dB per octave above 2.5 kHz (see electronic supplementary material, table S1, figure S1C,D). We used this simulated city-like noise for estimating masking of tutor songs and for playback.

### (e) Masking estimates

For all tutor songs, we assumed a song peak level of 80 dB for a communication distance of 1 m, which is typical for this species [25]. We set the noise playback at 54 dBA SPL, which is typical for noise levels in GGNRA [26]. We then quantified masking by city-like noise at three biologically relevant communication distances (7, 10 and 14 m, resulting in song peak SPL of 63, 60 and 57 dB, respectively) based on typical territory sizes in San Francisco (500 m^2^ (J.N.P. & R.M.D. 2015, unpublished data) which, if approximated as a circle, has a radius of 12.6 m). At each communication distance, we calculated the percentage duration of each tutor song that was masked by city-like noise by measuring the signal-to-noise ratio (SNR) of the peak frequency for each 5 ms of sound in the song and then determined for how many of these time periods the SNR was above the critical ratio in noise. For details, see electronic supplementary material, S1 and for examples of differentially masked tutor songs, see electronic supplementary material, figure S1. For background noise *L*_90_ see electronic supplementary material, S2 and for peak frequency SNRs for songs, see electronic supplementary material, S3.

### (f) Tutor song presentation

We presented juvenile males with a choice among songs that were differently masked by low-frequency noise to determine if males preferentially copy less-masked songs. Songs were sorted into two categories—lower-frequency songs that were more masked by noise or higher-frequency songs that were less masked—using methods described in ‘Masking estimates’ above. One song dialect (San Francisco [34,35]) included some songs that were categorized as more masked (recorded in the Twin Peaks neighbourhood) and some songs that were categorized as less masked (recorded in Battery East). Thus, the final set of tutor songs included more-masked, low-frequency song dialects (San Simeon, Manchester and Twin Peaks) and less-masked, high-frequency song dialects (Bonny Doon, Sonoma and Battery East). For each song category, we selected exemplars from three different males, as exposure to multiple renditions of a given song category may facilitate copy accuracy. We created eight different tutor sets. Each tutor set contained structurally distinct pairs of three renditions of a more masked dialect and three renditions of a less-masked dialect (e.g. three San Simeon songs versus three Bonny Doon songs) to facilitate tutor–tutee song matching. The tutor sets differed significantly in duration masked at each communication distance considered (paired *t*-test: 7 m: *t* = 26.5, d.f. = 7, *p* = 2.8 × 10^−8^; 10 m: *t* = 20.4, d.f. = 7, *p* = 1.7 × 10^−7^ (see electronic supplementary material, figure S1); 14 m: *t* = 12.8, d.f. = 7, *p* = 4.1 × 10^−6^) and the trilled portion of the song for less-masked, higher-frequency tutor songs was significantly higher in minimum and maximum frequencies as compared with high-masked tutor songs (*β* = 318.6 Hz min frequency, paired *t*-test, *t* = 2.315, d.f. = 7, *p* = 0.027 min; *t* = 3.528, d.f. = 7, *p* = 0.005 max). Tutor pairs did not differ in vocal performance—there were no significant differences in trill rate, frequency bandwidth or vocal performance (paired two-tailed *t*-tests: *p* > 0.2), nor did the actual tutor models copied by tutees (electronic supplementary material, table S2). We randomly assigned the eight tutor sets among the eleven noise-tutored males and then again among the six control males.

### (g) Song training

We used the same song training approach for the experimental and the control groups, and both groups heard the same eight combinations of low and high tutor songs—the difference being whether the noise was played at the same time as tutor songs (experimental) or at a separate time (control). Birds heard two bouts of each of their six tutor songs in a training session. Each tutor song was presented in a 5 min bout with songs repeated once every 10 s with silence between each song bout, for a total of 1 h of song training per session. We created 12 distinct patterns of playback of tutor songs to balance presentation of higher and lower frequency song types. The patterns randomized the initial song heard and the initial song category heard. Training files were programmed using Reaper v. 4.76 [33]. For both groups, we played stimuli using Windows PC running Reaper software through Saffire Focusrite 40 soundboards. The loudspeakers were Altec Lansing iM227 Orbit MP3 speakers that were approximately 12–30 cm from a bird, depending on where a bird was in their cage at any given time. The file format of tutor songs was .wav, 24 bit, 44.1 kHz.

We also live broadcast the city-like noise described above. Birds in the experimental group heard city-like noise simultaneously with playback of the tutor songs through the same speaker. We calibrated these playbacks to approximate a communication distance of 10 m (or approximately 7 m if factoring in excess attenuation in this habitat) between juvenile males and tutors (low-masked songs = 44.01 ± 3.24% (mean ± s.d.) duration masked, high-masked songs = 68.40 ± 0.68% duration masked). Thus, song playback was approximately 60 dB SPL and noise playback 54 dB SPL in the centre of a bird's cage. Because noise and song used the same speaker, the relative SNR is preserved when a bird moves around in its cage. Birds in the control group heard the city-like noise for the same length of time (one hour per training session) at a separate time from the broadcasting of tutor songs. Thus, birds in both groups were exposed to city-like noise for the same length of time to control for the corollary effects of noise such as stress [22,36].

We tutored twice daily (once in the morning and once in the afternoon), beginning near the start of the sensitive period for WCSP (NWCS subspecies typical sensitive period = 20–130 days; subjects average age = 23 days; range, 21–27 days) and continuing for 16 weeks (approximately 130 days of age). One training session per day (morning) was used for an additional 36 weeks, as this provided the only social stimulus received by the males. Tutoring stopped at the average age of 384 days, after all males produced crystallized songs and after all songs were recorded. All chambers were then opened to allow acoustic contact among subjects.

### (h) Sound analysis

All sound analyses made use of songs recorded during periods of time without concurrent noise playback. Sound spectrograms (256 pt transform, frequency resolution: 97.7 Hz, 10.2 ms time resolution) were made of 2–7 exemplars of each student song. Analysed song data are provided in electronic supplementary material, S4.

### (i) Statistical analysis of tutor song selection

We used two approaches to compare tutor song selection of noise-tutored males to that of control males. First, the songs were analysed qualitatively. Four treatment-blind researchers visually compared student songs to possible tutor songs to judge which tutor songs were copied. Comparisons took into account syllable morphology and note order. We compared tutor song selection between the two groups using an unconditional two-tailed Barnard's exact test under the binomial model.

We then quantitatively compared acoustic structure across a correlation matrix of all presented tutor songs and 2–7 renditions of each student's copies with spectrogram cross correlation (SPCC) analysis in SIGNAL v. 5 [31]. This technique quantifies the similarity of two signals with respect to duration, absolute frequency, modulations of frequency and amplitude. A pairwise comparison score of 1 indicates that two signals are identical. SPCC calculations were performed both with and without normalizing for frequency offset, on spectrograms constructed with 128–point fast Fourier transformations (FFTs) and 100 time steps, within a frequency range of 1.5–10 kHz. The results from the two methods were similar and we report values without frequency-shifting to be conservative. We performed this technique with whole songs for all birds; however, some birds failed to copy all parts of the song (e.g. produced only a whistle and trill, but left out complex and intermediate notes). For these six individuals, we broke their songs into their basic elements, selected each section of the song—whistle, whole trill, first and last trill syllables, first and last trill notes, buzz, and complex syllables—and compared each of these to each corresponding element of the six presented tutor songs. We averaged the highest match for each element copy to the corresponding element type in the tutor songs of each tutor set per individual, and these element-averages to each tutor group (more versus less masked) were used in place of the value for the whole-song SPCC. After identifying the maximum SPCC value quantifying the best match of each male's copied song to each category of tutor song (the high-frequency, less-masked tutor set and to the lower frequency, more masked tutor set), we performed a two-factor repeated-measures ANOVA with treatment group (control versus noise-tutored) and tutor category (more- versus less-masked tutor song) as the factors and bird identity as the repeated measure. We then performed post-hoc Tukey's tests to compare within treatment groups whether the copied songs better matched one tutor set or another. We performed these tests to determine if each experimental and control male's songs were better matched to one set of tutor songs than the other set.

### (j) Statistical analysis of song reproduction

For comparisons of acoustic structure between training models and student copies, we chose three high-quality exemplars of each tutee male's song, which were recorded during time periods without noise. We took measurements of the song minimum, peak, maximum frequencies and frequency bandwidth, and compared these to the same values for the copied tutor's song using two-factor, repeated measures ANOVA with song rendition (tutor versus copy) as the repeated measure. Songs were high-pass filtered at 1500 Hz to remove noise below the range of WCSP songs. A custom macro in SIGNAL measured frequencies from the power spectrum [37] with a 100 Hz smoothing width using 32 kpts total. The macro then normalized peak frequency to 0 dB and set a threshold of −36 dB from which to measure minimum and maximum frequencies. By graphing the amplitude power spectrum, we took these measurements for both whole songs and for the trilled portion of songs alone. Values for each variable were averaged across the three renditions for each male and log transformed.

Using log-transformed values, we performed a principal component analysis (PCA) decomposing minimum, peak and maximum frequencies into the first principal component which had an Eigenvalue greater than 1 (1.351) and explained 61% of the variation (electronic supplementary material, table S3). All variables satisfied the Shapiro–Wilks test for normality (except for log peak frequency) and Levene's test for equal variances. Therefore, we ran parametric analyses on all variables except for peak frequency. As a non-parametric alternative for peak frequency, we used a non-paired Wilcoxon rank sum test to compare songs from the 11 noise-tutored males and 6 control males. For PC1 (frequency), we conducted a two-factor repeated measures ANOVA testing treatment (control versus noise-tutored) and song rendition identity (tutor model versus tutee's copy), this time with song type rendition as the repeated measure (i.e. Battery East song 1: tutor and copy). To assess pairwise comparisons, we performed a post-hoc Tukey's test of contrasts by building a mixed-effects linear model with generalized linear model hypotheses, which gave *Z* statistics and *p*-values for all comparisons. We measured both whole songs and the trilled portion of songs, and all outcomes were in the same direction and similarly achieved or failed to achieve significance; therefore, we report our findings for trills alone, as the majority of information (complexity) occurs in the trilled portion of the song, whereas whistles are generally less-masked. All statistical analyses were conducted in R v. 3.3.0 [38].

### (k) Statistical analysis of song performance

We calculated vocal deviation for student and tutor songs. Vocal deviation is a measure of performance of a tradeoff between singing repeated syllables, ‘trills’, at rapid trill rates while maximizing frequency bandwidth [39]. This measure of performance is a salient feature of song in WCSP [40,41]. Trill rate was calculated as the average number of notes produced per second (Hz) for the terminal trill. To calculate frequency bandwidth, we subtracted the minimum frequency from the maximum frequency using the power spectrum as described above. We then calculated the orthogonal deviation of each song from an upper bound regression of trill bandwidth on trill rate (hereafter, vocal deviation [39]). We used the published equation for the upper bound regression on a set of 1572 songs that includes emberizids, *y* = −0.124*x* + 7.55 [37]. For frequency bandwidth and vocal deviation, we ran a two-factor repeated measures ANOVA with Tukey's mixed-effects models as described above for PC1.

## 3. Results

### (a) Noise-tutored males selected less-masked songs to copy

Noise exposure did not prevent species-typical song learning. All noise-tutored and control males (*n* = 17) produced a crystallized song, although one control male produced the elements of the copied song type out of order. All males produced a song type that could be matched to a tutor song (e.g. figure 1).

We assessed song matching both qualitatively using blind observers and quantitatively using spectrographic cross correlation (SPCC) in SIGNAL v. 5 [31]. Four observers blind to treatment and tutor category agreed on the song type copied by tutees, and our SPCC quantitative assessment was consistent with these assignments for every male. As predicted, noise-tutored and control males showed differences in selecting a song category to copy (Barnard's exact test: Barnard's CSM = 0.014, two-tailed *p* < 0.0396); 82% of noise-tutored males (9 out of 11) copied less-masked, higher-frequency songs. Notably, this result does not seem to derive from any intrinsic bias for copying high-frequency songs; this is illustrated by the learning outcomes in our control males, for which 67% of control males (four out of six) instead copied the lower-frequency songs (figure 1*a–h*).

In our follow-up quantitative assessment (SPCC), noise-tutored males' songs were significantly better matches of the less-masked tutor songs than the more-masked tutor songs (two-factor repeated-measures ANOVA: *F* = 4.358 *p* < 0.05, Tukey's post-hoc pairwise comparison within noise-tutored group of tutor category: *Z* = −2.952 *p* = 0.019; electronic supplementary material, table S4). By contrast, control males showed no significant difference between less-masked and more-masked tutor songs in their best match (Tukey's post-hoc pairwise comparison of tutor category within the control group, *Z* = −1.861 *p* = 0.3762; electronic supplementary material, table S4). These qualitative and quantitative assessments provide consistent evidence that noise-tutored males copied less-masked songs, whereas control males did not exhibit a copying preference.

### (b) Males exposed to masking noise reproduced copies at higher frequencies than their tutors

Next, we assessed whether copy quality (accuracy) varied between the noise-tutored and control males, and if males from both treatments reproduced their tutor's song at the original song frequencies. Control and noise-tutored males did not appear to differ in their ability to reproduce an accurate copy of tutors' songs (SPCC: 0 = no accuracy, 1 = perfect accuracy; average maximum copying accuracy control: 0.74 ± 0.02, experimental: 0.75 ± 0.03, mean ± standard error of the mean (s.e.m.)). However, treatments did differ in how they reproduced the acoustic structure of their tutors’ songs.

In our PCA of the structure of learned songs, peak and minimum frequencies loaded most strongly (0.68–0.69) and negatively onto the first principal component (PC1: eigenvalue = 1.35, variance explained = 0.61; electronic supplementary material, table S3). There was a significant effect of tutor model versus copy on PC1 frequency (two-factor repeated measures ANOVA: *F* = 7.919, *p* = 0.013; figure 1*i,j*, electronic supplementary material, table S2), such that noise-tutored males' songs differed significantly in frequency from the tutor dialect they copied (Tukey's contrasts: *Z* = 2.730, *p* = 0.038; figure 1*j*; electronic supplementary material, tables S2 and S4). Noise-tutored males reproduced their songs at significantly higher frequencies than their tutor song models (*β*_songs_ = +473.9 Hz minimum, *β*_trills_ = +425.9 Hz peak frequency; see electronic supplementary material, table S5 for frequency means and s.e.m.). By contrast, control males' songs did not differ in PC1 frequency from their tutors’ songs (Tukey's contrasts: *Z* = 1.040, *p* = 1.000; figure 1*i*; electronic supplementary material, table S2). We did not find an overall pattern of noise-tutored males singing higher-frequency songs on average than control males, probably because 33% of control males also copied high-frequency tutor songs. However, if we compared copies reproduced by noise-tutored to those by control males, we found that noise-tutored males sang their copies at higher average peak frequencies as compared with control males (*β*_trills_ = +307.9 Hz, Wilcoxon rank sum test *W* = 68.5, *p* = 0.023). Means, s.e. and residuals are presented in electronic supplementary material, table S5. These findings provide evidence that noise-tutored males reproduced their tutors' songs at higher minimum and peak frequencies whereas control males did not.

### (c) Noise-tutored males reproduced songs at lower vocal performance than their tutors

Finally, we examined the effects of masking noise during song tutoring on ‘vocal performance’, a song attribute that describes the ability to sing physiologically challenging songs well [42]. When comparing the copy to the tutor song, there was a significant effect for frequency bandwidth (two-factor repeated-measures ANOVA *F* = 25.5, *p* = 1.44 × 10^−4^; electronic supplementary material, figure S2) and for vocal performance measured as vocal deviation (figure 1*k,l*, electronic supplementary material, table S2, *F* = 13.2, *p* = 0.002). Specifically, noise-tutored males' trills had significantly narrower frequency bandwidths than the tutor song rendition that they copied (*β* = −822.5 Hz, Tukey's pairwise contrasts *Z* = 5.17, *p* = 1.4 × 10^−6^), which resulted in significantly lower overall vocal performance (figure 1*l*; vocal deviation *β* = 0.799, Tukey's pairwise contrasts *Z* = −3.332, *p* = 0.005). Despite the fact that noise-tutored males reproduced songs with reduced frequency bandwidths, their trill rate remained faithful to the tutor model (copies mean ± SD: 9.8 ± 2.0 Hz, tutors: 9.7 ± 2.6 Hz). Though not statistically significant, control males' trills also tended to have narrower-frequency bandwidths than their tutors’ trills and the effect size was smaller in magnitude (*β* = −322.9 Hz, *Z* = 1.50, *p* = 0.803; electronic supplementary material, tables S2 and S5). A slightly narrower bandwidth did not significantly impact the vocal performance of control males' songs compared with their tutors' songs (figure 1*k*; vocal deviation *β* = 0.518, *Z* = −1.60, *p* = 0.665), and there was no effect of treatment on the pairwise comparison of control and noise-tutored copies. In sum, noise-tutored males' selections and reproductions of tutor songs, but not control males’ reproductions of songs, negatively affected the vocal performance of learned songs.

## 4. Discussion

Our findings provide strong experimental support for cultural selection as a potential mechanism for acoustic adaptation to environments with anthropogenic noise. By copying the high-frequency, less-masked songs, noise-tutored males maximize the potential transmission and efficacy of their adult, crystallized songs in a noisy environment. The fact that a significant majority (82%) of noise-tutored males copied high-frequency tutor songs suggests strong selection acting on the avoidance of masking. Alternatively, males may follow cognitive tendencies to copy the most easily detected song [13], although 2 out of 11 noise-tutored males reproduced clear copies of the more-masked, lower-frequency songs, indicating that males could detect these tutor songs. Under either interpretation, these decisions in tutor song selection reveal how a song-learning bird species can increase signal transmission and avoid masking effects of noise through song selection across generations.

Our results differ from the one other experimental test of Hansen's hypothesis in a noisy environment, which did not find support for the reproduction of tutor songs at higher minimum frequencies in noise [20]. In addition to different focal species, a critical difference between our studies was that we collected individuals from loud, urban areas, whereas Zollinger *et al.* [20] collected from quiet, rural areas. The difference in natal environment raises the possibility that prior experience of noise may prime selectivity during song learning. This difference is analogous to findings in white-crowned sparrows [43] and in other species such as chickadees [44] in which individuals that breed in noisy environments vocally adjust to experimental noise whereas individuals in quieter environments do not. City-like noise was also twice as loud (+6 dB) in the study of Zollinger *et al*. [20], which potentially may have generated more of a stress response in both groups of their study subjects. However, because of differences in the noise spectral profile between our studies, their tutor songs were not necessarily more masked under the city noise treatment, based on data reported [45]. By contrast, all of our tutor songs were masked to some degree by noise. Thus, it is possible that our studies actually do not conflict; instead, Zollinger *et al.* [20] may not have found an effect of noise on vocal learning because their tutor songs were not differentially masked by city noise. Another study, which was not a test of the Hansen hypothesis, found no differences in song copying accuracy between quiet and noise-reared groups of zebra finches but did report differences in song syntax for a subgroup of birds, which may be an outcome of a chronic stress response to noise [46].

Our findings also suggest a second mechanism that results in reduced masking. Noise-tutored males not only copied higher-frequency songs but also all but one reproduced their learned songs at significantly higher frequencies than the original tutor song. Additionally, songs of noise-tutored males had higher peak frequency than songs of control males, even though one-third of control males also copied high-frequency tutor songs, indicating that noise-tutored males allocated more energy towards higher frequencies. Evidence of noise-tutored birds calibrating their tutor models during development indicates a mechanism distinct from cultural selection to reproduce songs at a higher frequency to increase signal transmission. This mechanism is also distinct from that of immediate flexibility, in which adult males shift song minimum frequency and other song traits in real time in response to changes in noise levels [9]. Calibration of minimum frequency in ontogeny may be the first indication of a potentially adaptive strategy in the context of refining vocal output to a specific environment and bears further examination in other species. However, below we discuss the potential costs of raising minimum frequency in a sexual selection framework.

Cultural evolution has been highlighted as having particular importance in affecting evolutionary change as a mechanism in species that learn because it enables rapid and immediate response to novel environmental pressures [47], such as anthropogenic noise [1]. New cultural traits can spread horizontally as well as vertically and, in some cases, faster than genetic traits [17]. Cultural evolution through social learning is widely regarded as the mechanism by which humans came to occupy such vast and distinct habitats [48]. Thus, cultural selection may also allow song-learning birds (as well as other species that learn their vocalizations) to adjust to habitats increasingly impacted by human-generated noise. The process of cultural selection over generations, potentially augmented by the adjustment of minimum frequency in ontogeny, could contribute to a population-level evolutionary trajectory towards songs with higher minimum frequencies. In fact, the minimum frequency of WCSP songs in San Francisco has increased both within and between dialects over a 30-year time span, and the dialect with the highest minimum frequency has nearly replaced another lower-frequency dialect within city limits [35]. We suggest that this observed pattern of temporal variation in song is consistent with a process of cultural evolution by selection of less-masked songs.

Adjusting the minimum frequency of songs to avoid noise may have functional consequences in a sexual selection context. Previous work shows that urban WCSP males respond to variation in song minimum frequency in territorial contexts [27]. However, follow-up studies found territorial males are not responding specifically to increases in song minimum frequency in noise but to the resulting reduction in bandwidth and vocal performance [41,49]. Vocal performance describes the ability to produce any song features that encounter physiological limits, such as rapid trills at wide-frequency bandwidths—two features that are known to trade off in emberizid sparrows [37] and other bird species [42]. In many species of songbirds, songs that maximize trill rate and frequency bandwidth are preferred by females [50–52], are sung by older, heavier males [53,54], and function as indicators of aggressive threat in male territorial disputes [55,56], including in WCSP [40,41]. Noise-tutored males were potentially limited in bandwidth because they sang higher minimum frequencies and probably encountered constraints in the maximum possible frequency [57,58]. A potential sexually selected pressure to sing at wider bandwidths may explain why WCSP control males in our study tended to copy lower frequency tutor songs, as lower minimum frequencies allow a wider possible bandwidth. We should note that noise-tutored and control males' copied songs did not differ significantly in absolute values of vocal performance, as we have measured a change in one experimental ‘generation’ to the next. Noise-tutored birds, however, produced copies of their tutor songs that were significantly lower in performance, while control males matched the performance of their tutors. If WCSP females assess vocal performance, as in other closely related species [50], then males with narrower bandwidths (and thus lower performance) could have reduced opportunities to attract mates and reproduce. Therefore, males in noisy environments may face a dilemma between maximizing signal transmission and maximizing vocal performance.

## 5. Conclusion

Our research contributes to the understanding of how noise pollution affects wild animals, especially those that persist in human-dominated landscapes. Numerous studies have shown the correlation between populations of animals living in noisy habitats vocalizing at higher frequencies [9]. Here, we provide strong experimental evidence of cultural selection as a potential mechanism to explain this pattern of directional selection on vocalizations. The combination of preferentially copying less-masked tutor songs and singing these songs at higher frequencies demonstrates the impact of ontogenetic exposure to masking noise on a species which relies on acoustic communication. Cultural evolution may be a critical strategy to mitigate the impacts of noise pollution, at least for animals that learn their acoustic signals.

## Supplementary Material

ESM 1: Supplementary Materials, Tables and Figures

## Supplementary Material

ESM 2: Background noise L90 values

## Supplementary Material

ESM 3: Peak frequency SNRs for tutor songs

## Supplementary Material

ESM 4: Song data

## Data accessibility

All data used in analyses are available in table format in electronic supplementary material, S2–S4 and on Dryad: http://dx.doi.org/10.5061/dryad.856rc00 [59].
