# Supplementary material for: Acoustic adaptation to city noise through vocal learning by a songbird
Source: Proc Biol Sci. 2018 Oct 10;285(1888):20181356. doi: 10.1098/rspb.2018.1356 (PMC6191693; doi:10.1098/rspb.2018.1356)
Supplement: ESM 1: Supplementary Materials, Tables and Figures [file rspb20181356supp1.docx]

**Title**: Acoustic adaptation to city noise through vocal learning by a songbird

**Authors**: D. L. Moseley^1,2,3^*, G. E. Derryberry^5^, J. N. Phillips^1^, J. E. Danner^1^, R. M. Danner^1,7^, D. A. Luther^2,4^, and E. P. Derryberry^1^*

**Journal:** Proceedings of the Royal Society of London Series B

**DOI:** 10.1098/rspb.2018.1356

**Contents**:

Methods. Hand-rearing methods

Methods. City-like noise generation

Methods. Masking estimates.

Table S1. Masking-noise filters

Table S2. ANOVAs and Tukey’s tests for tutor model and copy comparisons

Table S3. Principal Component Analysis

Table S4. ANOVA and Tukey’s test for Spectrographic Cross Correlation measures of copying accuracy

Table S5. Means and errors for response variables

Figure S1. Effects of city-like noise masking

Figure S2. Differences among groups in frequency bandwidth

**Methods.** Hand-rearing methods

Briefly, we fed birds by hand at half hour intervals from dawn to dusk until 10–12 days post hatch, then at 1–hour intervals until 18 days post hatch, and thereafter at 3-hour intervals until the birds were feeding independently at about 4–5 weeks of age. Young birds were hand-reared using the Marler diet [1] delivered from 1–cc syringes. Older birds were fed dry seed and water ad libitum, along with greens, soaked seed, hard-boiled eggs and a vitamin supplement. Males were individually housed in sound attenuation chambers (Industrial Acoustics Model Mac-1). Chamber dimensions were 68.6cm wide x 53.3cm deep x 63.5cm high (outside) and 58.4cm x 40.6cm x 35.6cm (inside). Each chamber contained a light, a fan for ventilation, and a loudspeaker (Altec Lansing iM227 Orbit MP3). Birds were kept on a natural photoperiod for San Francisco, controlled by time clocks (Hydrofarm TM01715D) that were changed once per week. The ambient temperature was maintained at 23°C. Within the chamber, males were housed in cages that measured 48.5 x 31 x 26 cm.

**Methods.** City like noise

We recorded two minutes of background noise using a Sennheiser ME62 omnidirectional microphone mounted facing upwards on a 1m tripod. We simultaneously measured the maximum sound pressure level every 10s using a tripod mounted 407736 Extech Sound Level Meter (response time = 125ms, accuracy = ±1.5dB, weighting = A). We calibrated the noise spectrum with the paired sound pressure levels using the Sound Level Meter function in SIGNAL, dropping outliers. We dropped outliers because the goal was to find the calibration constant for each background noise recording. Short temporal events (e.g., a dog bark or a person shout) can bias calibration. We determined outliers using a standard method based on quartiles. This was Q2 ± 1.5 * (Q3-Q1). To limit any bias in the calibration, we dropped identified outliers from both the recording and the SPL estimates for the calibration. We then averaged these 16 noise spectra and generated a noise file in Reaper 4.76 [2] to mimic this noise spectrum by applying an FFT filter to white noise, which decreased the spectral energy by 6 dB per octave up to 2.5 kHz and 9 dB per octave above 2.5kHz (see Table S1, Fig. S1 C, D).

**Methods.** Masking estimates

For all tutor songs, we calculated the total duration of song masked by city-like noise. We used the above noise spectrum and applied a masking function to determine the critical threshold (i.e., the amplitude of noise that would mask other sounds) at each frequency band. A masking function describes the signal-to-noise ratio (SNR) in decibels that is necessary for sound to be heard over background noise. We used a masking function (i.e., critical ratio) measured empirically for a closely related species, the song sparrow (*Melospiza melodia*) [3] because there is not a function specific to WCSP. We interpolated critical threshold values with a spline function between 1 and 8 kHz. Using the noise profile and masking function, we generated a masking profile by taking the higher of either the absolute threshold of the audibility curve or the critical threshold for the range of frequencies (1 to 8 kHz) in order to quantify the effects of the noise profile on how birds hear the tutor songs.

We calculated a spectrogram for each tutor song and for a 10sec long segment of city like noise (see above) with a time increment of 5 ms and a 256 pt FFT with a rectangular window (hereafter, a 'segment'). For each frequency in the noise spectrogram, we found the SPL exceeded 90% of the time (L_90_), and this was the background spectrum we used for masking calculations (see ESM 2). For each tutor song, we set the song peak level at 80 dB and assumed this as the level for a communication distance of 1m, which is typical for this species [4]. We measured the overall sound level for each segment. Segments more than 30 dB below the overall peak sound level for the whole song were denoted as 'silent'. For segments with sound, we first measured the level of the peak frequency (hereafter, 'peak level'). We determined the threshold sound level for the peak frequency using the masking profile. We then calculated the level of the peak frequency detected at a given communication distance using the masking profile. We offset the peak level using the communication distance formula for spherical propagation of sound (–20dB x (log_10_communication distance/1m); e.g., reduce by 20 dB for a 10m communication distance), which indicated the detected peak level. If the detected peak level was less than the threshold value that sound segment was denoted 'masked'. From this, we calculated the percentage of sound segments masked by city-like noise for a given communication distance. We repeated this estimate of masked song duration for three biologically relevant communication distances (7, 10 and 14m, which are 3 dB offsets) based on typical territory sizes for NWCS holding territories in San Francisco (500 m^2^ (unpublished data) which, if approximated as a circle, has a radius of 12.6m). See Fig. S1. To facilitate comparison with other studies, we also provide these data in the format of peak frequency SNR (peak SPL – the noise L_90_) for each 5ms time segment of each tutor song for the 10m communication distance, which is the distance used for determining playback sound levels (see ESM 3).

**Table S1.** Description of two filters applied in Reaper to simulate city-like noise. One filter generated white noise. The second filter decreased the spectral energy by 6dB per octave up to 2.5kHz and 9dB per octave above 2.5kHz.

| **1/3 octave bands** | **Spectral volume adjustment (dB)** | **Frequency** |
| --- | --- | --- |
| 14 | -48.06 | 23 |
| 21 | -18.25 | 123 |
| 24 | -12.13 | 246 |
| 27 | -6.03 | 498 |
| 30 | -0.05 | 1007 |
| 31 | 0 | 1259 |
| 32 | -1.65 | 1582 |
| 33 | -3.87 | 2003 |
| 34 | -5.84 | 2512 |
| 35 | -8.81 | 3162 |
| 36 | -12 | 3981 |
| 37 | -15 | 5012 |
| 38 | -18 | 6310 |
| 39 | -22 | 7943 |
| 40 | -26.25 | 10000 |

**Table S2.** ANOVA tables and posthoc Tukey’s test for examining how males’ copies compared to their selected tutor song. Two-way repeated measures ANOVA indicated that there was a significant effect of song identity (tutor model versus copy), and the posthoc test determined that noise-tutored (experimental) male’s copies significantly differed in their factor scores for (A) PC1 frequency, (B) frequency bandwidth, and (C) vocal deviation.

1. **PC1 Frequency**

ANOVA

| PC1 | DF | Sums of Squares | Mean Squares | F | p-value |  |
| --- | --- | --- | --- | --- | --- | --- |
| Group (con vs exp) | 1 | 4.280 | 4.281 | 1.823 | 0.197 |  |
| Residuals | 15 | 35.210 | 2.348 |  |  |  |
| **Song Identity (tutor model vs copy)** | **1** | **6.984** | **6.984** | **7.919** | **0.013** | * |
| interaction: Group * Song ID | 1 | 0.544 | 0.544 | 0.617 | 0.444 |  |
| Residuals | 15 | 13.229 | 0.882 |  |  |  |

Tukey’s Test

| PC1 Frequency (min, peak, max) |  |  |  |  |
| --- | --- | --- | --- | --- |
| Tukey's pair-wise comparisons | estimate | std.error | Z statistic | p-value |
| experimental.subject - control.subject | -1.007 | 0.645 | -1.562 | 0.710 |
| control.tutor - control.subject | 0.564 | 0.542 | 1.040 | 1.000 |
| experimental.tutor - control.subject | 0.086 | 0.645 | 0.133 | 1.000 |
| control.tutor - experimental.subject | 1.571 | 0.645 | 2.436 | 0.089 |
| **experimental.tutor - experimental.subject** | **1.093** | **0.400** | **2.730** | **0.038** |
| experimental.tutor - control.tutor | -0.478 | 0.645 | -0.741 | 1.000 |

1. **Frequency Bandwidth**

ANOVA

| Bandwidth | DF | Sums of Squares | Mean Squares | F | p-value |  |
| --- | --- | --- | --- | --- | --- | --- |
| Group (con vs exp) | 1 | 12314 | 12314 | 0.016 | 0.901 |  |
| Residuals | 15 | 11590470 | 772698 |  |  |  |
| **Song Identity (tutor model vs copy)** | **1** | **3548576** | **3548576** | **25.496** | **1.44E-04** | *** |
| interaction: Group * Song ID | 1 | 484486 | 484486 | 3.481 | 0.082 | . |
| Residuals | 15 | 2087731 | 139182 |  |  |  |

Tukey’s Test

| Frequency Bandwidth |  |  |  |  |
| --- | --- | --- | --- | --- |
| Tukey's pair-wise comparisons | estimate | std.error | Z statistic | p-value |
| experimental.subject - control.subject | -289.615 | 342.693 | -0.845 | 1.000 |
| control.tutor - control.subject | 322.867 | 215.394 | 1.499 | 0.803 |
| experimental.tutor - control.subject | 532.835 | 342.693 | 1.555 | 0.720 |
| control.tutor - experimental.subject | 612.482 | 342.693 | 1.787 | 0.443 |
| **experimental.tutor - experimental.subject** | **822.450** | **159.079** | **5.170** | **1.40E-06** |
| experimental.tutor - control.tutor | 209.968 | 342.693 | 0.613 | 1.000 |

**C. Vocal Deviation**

ANOVA

| Vocal Deviation | DF | Sums of Squares | Mean Squares | F | p-value |  |
| --- | --- | --- | --- | --- | --- | --- |
| Group (con vs exp) | 1 | 0.176 | 0.176 | 0.208 | 0.655 |  |
| Residuals | 15 | 12.703 | 0.847 |  |  |  |
| **Song Identity (tutor model vs copy)** | **1** | **4.160** | **4.160** | **13.159** | **0.002** | ****** |
| interaction: Group * Song ID | 1 | 0.153 | 0.153 | 0.486 | 0.497 |  |
| Residuals | 15 | 4.741 | 0.316 |  |  |  |

Tukey’s Test

| Vocal Deviation |  |  |  |  |
| --- | --- | --- | --- | --- |
| Tukey's pair-wise comparisons | estimate | std.error | Z statistic | p-value |
| experimental.subject - control.subject | -0.010 | 0.387 | -0.025 | 1.000 |
| control.tutor - control.subject | -0.518 | 0.325 | -1.595 | 0.665 |
| experimental.tutor - control.subject | -0.809 | 0.387 | -2.089 | 0.220 |
| control.tutor - experimental.subject | -0.508 | 0.387 | -1.312 | 1.000 |
| **experimental.tutor - experimental.subject** | **-0.799** | **0.240** | **-3.332** | **0.005** |
| experimental.tutor - control.tutor | -0.291 | 0.387 | -0.752 | 1.000 |

**Table S3.** Three variables of song frequency were decomposed using principle component analysis. Loadings are reported for two principal components. The Eigen value of PC1 was greater than 1, thus we only used PC1 in our frequency analyses. Both log minimum and log peak frequency loaded strongly and negatively onto PC1.

|  | **PC1** | **PC2** |
| --- | --- | --- |
| Eigen value | **1.351** | 0.979 |
| Proportion of Variance | 0.609 | 0.319 |
|  |  |  |
| log minimum | **-0.693** | 0.117 |
| log maximum | 0.228 | 0.972 |
| log peak | **-0.684** | 0.206 |

**Table S4**. ANOVA tables and posthoc Tukey’s test for testing whether males’ copies were better matched to high or low frequency tutor sets. Copying accuracy is measured by Spectrographic Cross Correlation (SPCC), which provides a percent match to any tutor song. Two-way repeated measures ANOVA indicated that there was a significant effect of tutor set, and the posthoc test revealed that noise-tutored (experimental) males’ songs were better matches of the high-frequency, less-masked songs.

| **ANOVA** |  |  |  |  |  |  |
| --- | --- | --- | --- | --- | --- | --- |
| SPCC (percent match) | DF | Sums of Squares | Mean Squares | F | p-value |  |
| Group (con vs exp) | 1 | 0.011 | 0.011 | 1.457 | 0.237 |  |
| **tutor set (more vs less masked)** | **1** | **0.032** | **0.032** | **4.358** | **0.046** | ***** |
| interaction: group * tutor set | 1 | 0.027 | 0.027 | 3.613 | 0.067 | . |
| Residuals | 29 | 0.215 | 0.007 |  |  |  |

Tukey’s Test for all pair-wise comparisions. Abbreviations indicate the following:

| Group | Description |
| --- | --- |
| exp | noise-tutored males |
| con | control males |
| Tutor |  |
| high | match of student's copy to high frequency, less masked tutor songs |
| low | match of student's copy to low frequency, more masked tutor songs |

| SPCC (percent match) |  |  |  |  |  |
| --- | --- | --- | --- | --- | --- |
| Tukey's pair-wise comparisons | estimate | std.error | Z statistic | p-value |  |
| exp.high - con.high | 0.096 | 0.044 | 2.188 | 0.172 |  |
| con.low - con.high | 0.014 | 0.047 | 0.304 | 1.000 |  |
| exp.low - con.high | -0.007 | 0.044 | -0.160 | 1.000 |  |
| con.low - exp.high | -0.082 | 0.044 | -1.861 | 0.376 |  |
| **exp.low - exp.high** | **-0.103** | **0.035** | **-2.952** | **0.019** | ***** |
| exp.low - con.low | -0.021 | 0.044 | -0.487 | 1.000 |  |

**Table S5.** Means and errors for response variables for birds in control and experimental treatments and tutor models. Response variables include, (A) minimum frequency, (B) peak frequency, (C) maximum frequency, (D) frequency bandwidth, and (E) vocal deviation.

A. Minimum Frequency

| group | song identity | N | Minimum Frequency | sd | se | ci |
| --- | --- | --- | --- | --- | --- | --- |
| control | copy | 6 | 2978 | 516 | 211 | 542 |
| control | tutor model | 6 | 2830 | 452 | 185 | 474 |
| experimental | copy | 11 | 3268 | 564 | 170 | 379 |
| experimental | tutor model | 11 | 3019 | 294 | 89 | 197 |

B. Peak Frequency

| group | song identity | N | average peak | sd | se | ci |
| --- | --- | --- | --- | --- | --- | --- |
| control | copy | 6 | 4194 | 441 | 180 | 463 |
| control | tutor model | 6 | 4088 | 385 | 157 | 404 |
| experimental | copy | 11 | 4501 | 341 | 103 | 229 |
| experimental | tutor model | 11 | 4203 | 303 | 91 | 204 |

C. Maximum frequency

| group | song identity | N | avg. max | sd | se | ci |
| --- | --- | --- | --- | --- | --- | --- |
| control | copy | 6 | 6078 | 618 | 252 | 649 |
| control | tutor model | 6 | 6385 | 288 | 118 | 303 |
| experimental | copy | 11 | 6086 | 545 | 164 | 366 |
| experimental | tutor model | 11 | 6556 | 331 | 100 | 222 |

D. Frequency Bandwidth

| group | song identity | N | average bandwidth | sd | se | ci |
| --- | --- | --- | --- | --- | --- | --- |
| control | copy | 6 | 3100 | 1044 | 426 | 1096 |
| control | tutor model | 6 | 3423 | 876 | 358 | 920 |
| experimental | copy | 11 | 2811 | 563 | 170 | 378 |
| experimental | tutor model | 11 | 3633 | 348 | 105 | 234 |

E. Vocal Deviation

| group | Song identity | N | avg. vocal deviation | sd | se | ci |
| --- | --- | --- | --- | --- | --- | --- |
| control | copy | 6 | 3.50 | 1.14 | 0.463 | 1.19 |
| control | tutor model | 6 | 2.99 | 0.869 | 0.355 | 0.912 |
| experimental | copy | 11 | 3.49 | 0.730 | 0.220 | 0.490 |
| experimental | tutor model | 11 | 2.69 | 0.436 | 0.132 | 0.293 |

**
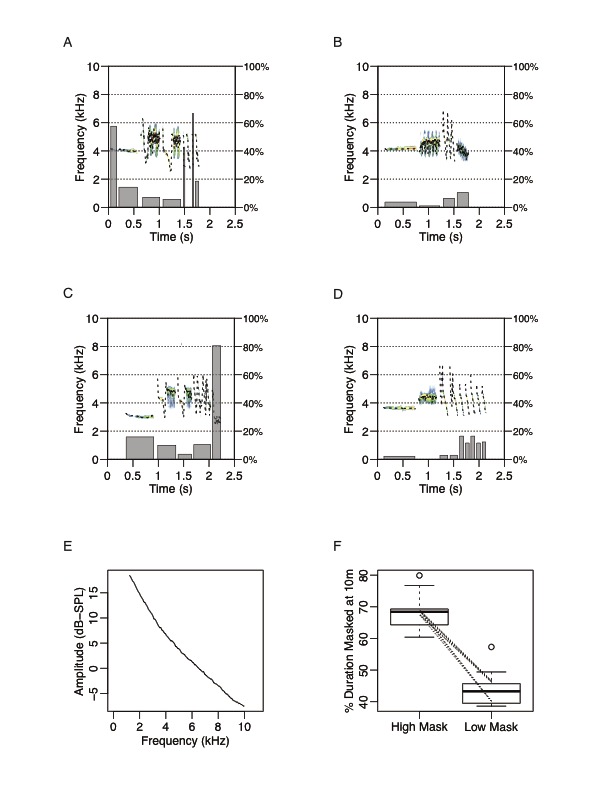
**

**FIGURE S1. Effects of city-like noise masking**

Example spectrograms A-D correspond directly to spectrograms A-D in Figure 1 and depict frequency (kHz) by time (s) on the left and percent masking by noise on the right Y axis. A and C represent lower frequency tutor songs that were more highly masked by noise, B and D represent higher frequency tutor songs that were less masked by noise. Bar plots on top of each spectrogram indicate the percentage each note type that was masked by noise. Noise-tutored subjects were trained with eight different combinations of lower and higher frequency songs of which A & B and C & D are two example combinations. Each male heard three different renditions, which were wild-recorded from three different males, of each tutor dialect, i.e. three renditions of A and three of B, for example. Control male subjects were trained with the same combinations but heard noise at a different time of day, separate from song tutoring.

(E) The amplitude spectrum of the masking noise, which is a rolling function similar to anthropogenic noise recorded at our Battery East field site in GGNRA. Decibel sound pressure level is graphed against frequency (kHz).

(F) Songs categorized as high masked (low frequency) significantly differed from songs categorized as low masked (high frequency) in the percent duration masked by noise, as measured at 10m. Box plot: bold middle lines indicate the median, bottom and top of the box borders indicate 25th and 75th percentiles, whiskers represent the minimum and maximum, and circles represent outliers.


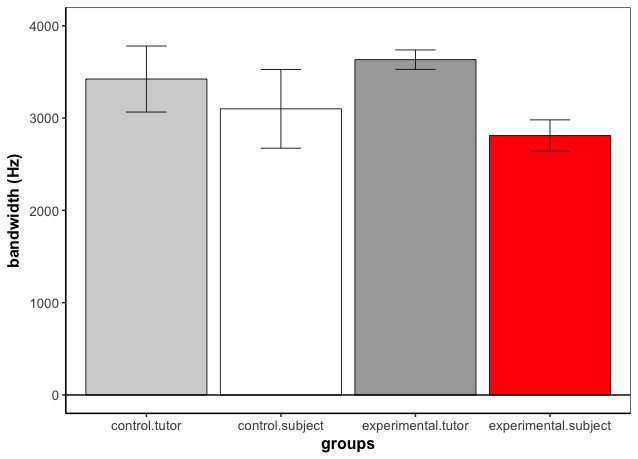


**FIGURE S2. Differences among groups in frequency bandwidth**

Noise-tutored males differed in the frequency bandwidth of their copies as compared to the exact tutor model they copied (Tukey’s pairwise comparison. There was an effect of copy versus tutor song on frequency bandwidth (two-factor repeated measures ANOVA F = 25.5, *P* = 1.44*10^-04^, SI Table S5). Specifically, noise-tutored males’ trills had significantly narrower frequency bandwidths than the exact tutor song rendition that they copied (β = -822.5 Hz, Tukey’s pairwise contrasts Z = 5.17, *P* = 1.4e-06). Control males’ trills did not have significantly narrower frequency bandwidths than their tutors’ trills (Tukey’s pairwise comparison Z = 1.50, *P* = 0.803, SI Table S4, 5). In the two-way repeated measures ANOVA, there was trend for the interaction between treatment group and song identity (model vs copy), but did not reach statistical significance at alpha = 0.05 (F = 3.481 *P* = 0.082).

**References**

1 Searcy, W. A., Peters, S. & Nowicki, S. 2004 Effects of early nutrition on growth rate and adult size in song sparrows Melospiza melodia. *Journal of Avian Biology* **35**, 269-279

2 Cockos. 2016 Rapid environment for audio production, engineering and recording, vol. 4. San Francisco, CA.

3 Dooling, R. J. 2002 Avian hearing and the avoidance of wind turbines. (ed. N. R. E. Laboratory). Golden, Colorado: U.S. Department of Energy Laboratory.

4 Derryberry, E. P., Gentry, K. E., Derryberry, G. E., Phillips, J. N., Danner, R. M., Danner, J. E. & Luther, D. A. 2017 White-crowned sparrow males show immediate flexibility in song amplitude but not in song minimum frequency in response to changes in ambient noise levels in the field. . *Ecology & Evolution* **7**, 4991-5001
